# Clinical epidemiology of Epstein-Barr virus-associated Lymphoproliferative Disorders (EBV-LPDs) in hospitalized children: A six-year multi-institutional study in China

**DOI:** 10.1186/s13052-024-01685-y

**Published:** 2024-07-02

**Authors:** Dilara Dilmurat, Xinyu Wang, Liwei Gao, Jiao Tian, Junhong Ai, Linlin Zhang, Mengjia Liu, Guoshuang Feng, Yueping Zeng, Ran Wang, Zhengde Xie

**Affiliations:** 1 Beijing Key Laboratory of Pediatric Respiratory Infectious Diseases, Key Laboratory of Major Diseases in Children, Ministry of Education, National Clinical Research Center for Respiratory Diseases, Laboratory of Infection and Virology, Beijing Pediatric Research Institute, Beijing Children’s Hospital, Capital Medical University, National Center for Children’s Health, Beijing, 100045 China; 2 Research Unit of Critical Infection in Children, 2019RU016, Chinese Academy of Medical Sciences, Beijing, 100045 China; 3 Big Data Center, Beijing Childre’s Hospital, Capital Medical University, National Center for Children’s Health, Beijing, 100045 China; 4 Department of Respiratory Medicine, National Center for Children’s Health, National Clinical Research Center of Respiratory Diseases, Beijing Children’s Hospital, Capital Medical University, Beijing, 100045 China; 5 Medical Oncology Department, Pediatric Oncology Center, Beijing Children’s Hospital, National Center for Children’s Health, Beijing Key Laboratory of Pediatric Hematology Oncology, Key Laboratory of Major Diseases in Children, Ministry of Education, National Key Discipline of Pediatrics, Capital Medical University, Beijing, 100045 China; 6 Medical Record Management Office, Beijing Children’s Hospital,Capital Medical University, National Center for Children’s Health, Beijing, 100045 China

**Keywords:** Epstein-Barr virus, EBV-LPD, Epidemiology, Hospitalization, Children

## Abstract

**Background:**

Epstein-Barr virus-associated lymphoproliferative disorders (EBV-LPDs) are a group of disorders involving lymphoid tissues or lymphocytes. The epidemiology and economic burden of hospitalized children with EBV-LPDs in China have not been well studied. This study aimed to reveal the epidemic characteristics and disease burden of EBV-LPDs among the Chinese hospitalized children, providing strategies for the prevention and management.

**Methods:**

This study was based on the FUTang Updating medical REcords (FUTURE) database of China and collected the medical records from 27 tertiary children’s hospitals between January 2016 and December 2021 in China, counting five types of EBV-LPDs, namely EBV-positive T-cell lymphoproliferative disease, NK/T cell lymphoma, extranodal NK/T-cell lymphoma (nasal type), systemic EBV-positive T-cell lymphoproliferative disease of childhood and posttransplant lymphoproliferative disorders. We conducted a retrospective syhthesis and analysis of the epidemiological characteristics, expenses, length of stay (LOS), as well as complications among hospitalized children diagnosed with five types of EBV-LPDs and compared parameters using appropriate statistical tests.

**Results:**

The study described 153 children aged 0–18 years hospitalized with EBV-LPDs from 2016 to 2021 in the FUTURE database. The male-to-female ratio was 1.10:1, and more than half of the age distribution was in the 6–12 y group. Among EBV-LPDs cases, EBV^+^ T-LPD accounted for the largest proportion (65.36%). Complications were presented in 93 children with EBV-LPDs, mainly hemophagocytic lymphohistiocytosis (HLH). The median LOS of NKTL was 26.5 days [interquartile range (IQR) = 3–42], which was the longest among EBV-LPDs. The median hospitalization cost of PTLD was 10 785.74 United States dollars (IQR = 7 329.38–16 531.18), which was the heaviest among EBV-LPDs.

**Conclusions:**

Compared with the total number of hospitalized children in China during the same period and in the same age group, the proportion of EBV-LPD is very low. EBV-LPD can develop in all age groups, but it is more common in school-age children. Among 5 EBV-LPDs, the disease with the highest proportion is EBV^+^ T-LPD. The overall disease burden of EBV-LPD was heavy, especially the economic burden. HLH was one of the most common complications, which could directly affect the burden of patients because of prolonged hospitalization. These data are taken from a very large database, illustrating the epidemiological and economic burden of EBV-LPDs hospitalized children in China, which enriched the existing epidemiological and disease burden content of EBV-LPDs.

**Supplementary Information:**

The online version contains supplementary material available at 10.1186/s13052-024-01685-y.

## Background

Lymphoproliferative disorders (LPDs) are a group of disorders characterized by immune system dysfunction, including both non-neoplastic and neoplastic disorders, and can be categorized into B-cell-associated LPDs and T/NK-cell-associated LPDs based on the type of lymphocyte proliferation [1]. Although LPDs are relatively rare compared to other diseases, the severity of LPDs is usually heavy and children with these disorders are susceptible to serious infection.

Epstein-Barr virus (EBV) is the first virus that is closely associated with human tumors, isolating from a Burkitt lymphoma cell line in 1964 [2]. There are approximately 200 000 new cancer cases attributed to EBV each year in worldwide, with LPDs comprising a significant portion [3]. In most immunocompetent individuals, EBV is latent in memory B cells and is controlled by a strong cytotoxic T lymphocyte response. However, especially in pathogenic settings, EBV can also infect T cells and natural killer (NK) cells, resulting in chronic persistent active infection [4, 5]. Many EBV-associated LPDs (EBV-LPDs) occur in children without immunodeficiency, and individuals with inherited disorders, infectious diseases (e.g., human immunodeficiency virus infection), or immunosuppression are also at high risk of developing EBV-LPDs [6].

EBV-LPDs cover a wide spectrum of diseases and is mostly common in Asia, especially East Asia [7, 8]. The general introduction of some EBV-LPDs is shown in Table 1 below. Once children are affected, the disease progresses rapidly and has a poor prognosis. In response to the severity of EBV-LPDs, both WHO and the Chinese government have implemented a range of intervention measures aimed at mitigating the disease burden associated with these conditions. Firstly, in addition to the numerous guidelines issued by WHO, the improvement of national medical standards and the formulation of guidelines and consensus documents have also provided strong guarantees for the prevention and treatment of EBV-associated diseases. Most importantly, the National Children's Medical Center (Beijing Children’s Hospital) established the National Center for Pediatric Cancer Surveillance to provide comprehensive support for the prevention and control of children's hematological malignancies [9]. Secondly, the government strongly supports community health education about EBV infection and encourages hospitals to conduct free clinic activities and other initiatives. Furthermore, the government have invested significantly in scientific research, including the development of EBV vaccines [10]. Despite these efforts, the specific clinical epidemiology and disease burden of EBV-LPDs in China have not been described in detail. Understanding the prevalence of EBV-LPDs is of significant public health importance. Therefore, this study aimed to reveal the epidemic characteristics and disease burden (e.g. hospitalization cost, complication rates, etc.) of EBV-LPDs among the Chinese hospitalized children based on a national pediatric patient database of China. We retrospectively analyzed the clinical epidemiology and disease burden-related data of Chinese hospitalized children diagnosed with five types of EBV-LPDs, namely EBV-positive T-cell lymphoproliferative disease (EBV^+^ T-LPD), NK/T cell lymphoma (NKTL), extranodal NK/T-cell lymphoma (nasal type) (ENKTL-NT), systemic EBV-positive T-cell lymphoproliferative disease of childhood (CSEBV^+^ T-LPD) and posttransplant lymphoproliferative disorders (PTLD), from 2016 to 2021. A particular strength of this study that could add to the current body of knowledge is the fact that the data are taken from a very large database representing diverse geographic areas of one of the largest countries in Asia. In general, the data provide a reference for clinicians to some extent.

**Table 1:** The general introduction of 5 EBV-LPDs

Classification	Disorders	High-incidence age	Incidence	Disease characteristics	Prognosis	EBV association
T- and NK- cell associated EBV-LPD	EBV^+^ T-LPD	usually occurs in children and young adults after primary EBV infection	A group of rare diseases, the incidence has not been reported, but it is more common in East Asia and Latin America[26]	A less common proliferative disorder of T lymphocytes. This disease has significant clinicopathological similarities to EBV-associated HLH[27]	overall poor prognosis and high case fatality rate	Acute EBV infection occurs in most cases
	NKTL	common in adults, but also reported in children	approximately 10% of NHL in Asia and South America, but only 1% in North America and Western Europe[28, 29]	In 2016 WHO guideline, NKTL is categorized as EBV-associated T/NK cell associated EBV-LPD, which can be either nodal or extranodal in type[29]	high invasive and poor prognosis	NKTLs are commonly infected by EBV[30]
	ENKTL-NT	common in adults, but also reported in children	only 0.2%–0.4% of NHL in the United States and Europe, while 5%–15% of NHL in Asia and Latin America[31–33]	This disease is characterized by the destruction of the midline facial structures, often manifesting as perforation of the hard palate[34]	high invasive and poor prognosis	EBV is detectable in almost all tumor cells[35]
	CSEBV^+^ T-LPD	This disease is generally rare, occurs mainly in children[36]	mostly prevalent in East Asia and Latin America[37]	Fever is the most common clinical manifestation, followed by abnormal liver function, splenomegaly and thrombocytopenia	Disease progresses rapidly and is deadly	Acute EBV infection occurs in most cases[38]
Immunodeficiency related EBV-LPD	PTLD	Appears in all age group	The incidence rate ranges from < 1% to 10% depending on the transplanted organ[39]	It is a lymphoproliferative disease caused by immunosuppression after solid organ transplantation or allogeneic hematopoietic stem cell transplantation (alloHSCT). It mostly occurs within 6 months after transplantation.[39, 40]	The prognosis is often poor with a high mortality rate, some cases are diagnosed post-mortem[39]	Almost all PTLD that occurred after alloHSCT was associated with EBV[41]

## Methods

### Data source

The Futang Research Center of Pediatric Development (FRCPD) is the first nonprofit social service organization engaged in pediatric development research under the supervision and management of the Ministry of Civil Affairs of China. FRCPD began collecting the face sheet of discharge medical records (FSMRs) in December 2015, and a total of 27 hospitals have reached an agreement to upload the FSMRs of hospitalized children to the FUTang Update medical REcords (FUTURE) database annually. All of these 27 hospitals are covering seven geographic regions of China: east, north, central, northeast, northwest, south and southwest China, among them, 21 of these hospitals are located in the provincial capital. The staff at FRCPD were responsible for checking and validating the uploaded data to control its quality and integrity. The data of this study came from the FUTURE database, and the FSMRs information of hospitalized children from 2016 to 2021 was extracted.

### Study variables

In this study, we extracted information on hospitalized children diagnosed with 5 types of EBV-LPDs in the FUTURE database. These five types of EBV-LPDs can be divided into two major categories [1]: T- and NK-cell-associated EBV-LPD and immunodeficiency related EBV-LPD. T- and NK-cell related EBV-LPD includes 4 types of diseases: EBV^+^ T-LPD, CSEBV^+^ T-LPD, NKTL, and ENKTL-NT. In addition, immunodeficiency related EBV-LPD includes PTLD.

We extracted relevant medical information such as gender, age, geographic regions, ethnicity, hospitalization time, diagnosis, length of stay (LOS), expenses, complications, history of blood transfusion for analysis. The general sociodemographic information is shown in Table 2. In age grouping, we searched for pediatric patients of all ages 0–18 y. Among them, hospitalized patients are divided into five groups according to their age (day, d, or year, y): 0–28 d (neonate), 29 d– < 1 y (infant), 1–3 y (toddler), 4–6 y (preschool child), 6–12 y (school-age child), and 13–18 y (adolescence). The patients included in the study were irrespective of gender or ethnicity. The data on complications were limited to those that occurred during the hospitalization for EBV-LPDs.

**Table 2:** The general sociodemographic characteristics of pediatric patients with EBV-LPD during hospitalization from January 2016 to December 2021

Categories	EBV-LPD (n, %)				
	**NK- and T-cell EBV-LPD**				**Immunodeficiency related EBV-LPD**
	**EBV**^**+**^** T-LPD**	**ENKTL-NT**	**CSEBV**^**+**^** T-LPD**	**NKTL**	**PTLD**
**Overall (n, %)**	100 (65.36)	27 (17.65)	11 (7.19)	6 (3.92)	9 (5.88)
**Gender (n, %)**					
Male	49 (49.00)	16 (59.26)	7 (63.64)	3 (50.00)	5 (55.56)
Female	51 (51.00)	11 (40.74)	4 (36.36)	3 (50.00)	4 (44.44)
**Age (n, %)**					
0–28 d	0 (0.00)	0 (0.00)	0 (0.00)	0 (0.00)	0 (0.00)
29 d – < 1 y	5 (5.00)	2 (7.41)	1 (9.09)	0 (0.00)	1 (11.11)
1–3 y	7 (7.00)	1 (3.70)	1 (9.09)	0 (0.00)	1 (11.11)
4–6 y	16 (16.00)	4 (14.81)	2 (18.18)	0 (0.00)	0 (0.00)
6–12 y	57 (57.00)	14 (51.85)	4 (36.36)	3 (50.00)	5 (55.56)
13–18 y	15 (15.00)	6 (22.22)	3 (27.27)	3 (50.00)	2 (22.22)
**Region (n, %)**					
Northeast China	0 (0.00)	0 (0.00)	0 (0.00)	1 (16.67)	0 (0.00)
North China	65 (65.00)	18 (66.67)	7 (63.64)	2 (33.33)	4 (44.44)
East China	13 (13.00)	6 (22.22)	3 (27.27)	0 (0.00)	1 (11.11)
South China	0 (0.00)	0 (0.00)	0 (0.00)	0 (0.00)	3 (33.33)
Central China	22 (22.00)	2 (7.41)	0 (0.00)	1 (16.67)	1 (11.11)
Northwest China	0 (0.00)	1 (3.70)	1 (9.09)	1 (16.67)	0 (0.00)
Southwest China	0 (0.00)	0 (0.00)	0 (0.00)	1 (16.67)	0 (0.00)
**Ethnicity (n, %)**					
Han	95 (95.00)	25 (92.59)	11 (100.00)	5 (83.33)	9 (100.00)
Non-Han	5 (5.00)	2 (7.41)	0 (0.00)	1 (16.67)	0 (0.00)
**Residence (n, %)**					
Urban	36 (36.00)	6 (22.22)	4 (36.36)	2 (33.33)	5 (55.56)
Rural	64 (64.00)	21 (77.78)	7 (63.64)	4 (66.67)	4 (44.44)
** LOS [d, median (IQR)]**	20.5 (10–37.75)	24 (9–34)	14 (8–33)	26.5 (3–42)	19 (11–32)
** Expense [USD, median (IQR)]**	6682.11 (2540.00–22,329.41)	8991.76 (2546.32–21,239.79)	7400.10 (2806.80–33,536.52)	8833.31 (1104.24–26,001.48)	10,785.74 (7329.38–16,531.18)

*LPD* Lymphoproliferative Disorders*, EBV*^+^ *T-LPD* EBV-positive T-cell Lymphoproliferative Disease*, ENKTL-NT* Extranodal NK/T Cell Lymphoma, nasal type*, CSEBV*^+^ *T-LPD* Systemic EBV-positive T-cell Lymphoproliferative Disorder of Childhood*, NKTL* NK/T cell Lymphoma*, PTLD* Posttransplant Lymphoproliferative Disorders*, EBV* Epstein-Barr Virus*, LOS* Length of stay*, IQR* Inter quartile range*, USD* USA dollar

### The eligibility of the participants and admission records

The inclusion criteria of the participants were as follows [11]:

1. All included patients who were admitted with the 5 EBV-LPDs mentioned above between January 1st, 2016 and December 31st, 2021. The 10th Revision of International Statistical Classification of Diseases and Related Health Problems (WHO, ICD-10) codes (https://icd.who.int/browse10/2016/en) was used as the selection criteria for the primary screening and classification of disease. According to the ICD-10 codes, we searched for hospitalizations with the first diagnosis of EBV^+^ T-LPD, ENKTL-NT, NKTL, CSEBV^+^ T-LPD and PTLD under the database.

The diagnostic criteria for the 5 diseases are as follows:

1. EBV^+^ T-LPD, CSEBV^+^ T-LPD, NKTL, ENKTL-NT: National guidelines for diagnosis and treatment of malignant lymphoma 2022 in China [12].
2. PTLD: Chinese consensus on the diagnosis and management of Epstein-Barr virus-related post-transplant lymphoproliferative disorders after hematopoietic stem cell transplantation (2022) [13].

The exclusion criteria of the participants were as follows:

1. Patients with multiple vital data incomplete, such as patient sex, age, or disease burden (LOS and expense), were excluded.
2. Cases with vague diagnosis were excluded.

### Statistical analysis

Categorical variables are described as percentages. The median with interquartile ranges (IQR) is used as a measure of central tendency. For nonordinal categorical variables, the chi-squared test was used to compare percentages among groups. For ordinal categorical variables and nonnormal distribution data, the nonparametric Wilcoxon or Kruskal–Wallis test was used for the comparison among groups. All statistics were analyzed by using SPSS software version 22.0 (SPSS Inc., USA). Differences with *p* values of < 0.05 were considered statistically significant.

## Results

### Overall

As shown in Table 2, a total of 153 patients were hospitalized for any one of the above 5 EBV-LPDs, and the total number of hospitalizations between 2016 and 2021 recorded in the database was 7 626 411. The overall male-to-female ratio of EBV-LPD was 1.10:1, and the age of onset was mostly among school-age children aged 6–12 years old (83, 54.25%). Among these 5 EBV-LPDs, EBV^+^ T-LPD cases accounted for the largest proportion (100 cases, 65.36%) followed by ENKTL-NT, CSEBV^+^ T-LPD, PTLD, and NKTL. Hospitalized children with NKTL had the longest LOS of 26.5 days (IQR 3–42) and an expenditure of 8 833.31 USD (IQR 1 104.24–26 001.48), while PTLD had the highest expenditure of 10 785.74 USD (IQR 7 329.38–16 531.18) and the LOS of 19 days [IQR 11–32]. Among the 153 patients, 93 were known to have complications. Table 3 shows the distribution of complications in different EBV-LPDs. Among complications, HLH had the highest incidence rate. We also conducted Fisher's exact test to compare whether there are differences in the same complication among the five EBV-LPDs listed in Table 3. However, the results indicated no significant difference.

**Table 3:** Distribution of complications in different EBV-LPDs

Categories	EBV-LPDs (*n*)				
	**EBV**^**+**^** T-LPD**	**ENKTL-NT**	**CSEBV**^**+**^** T-LPD**	**NKTL**	**PTLD**
**Complications (n)**					
** Overall (n)**	49	22	9	5	8
HLH	22	6	6	2	0
Digestive system disease (abnormal liver function, liver damage, gastrointestinal bleeding, intestinal infection)	12	1	0	1	1
Respiratory infection (pulmonary infection, pneumonia, bronchopneumonia, acute bronchitis, respiratory failure, etc.)	9	5	2	0	4
Chemotherapy induced myelosuppression	3	3	0	1	0
Sepsis	3	2	0	1	1
Cardiovascular system disease (myocardial strain, thrombotic microangiopathy)	0	2	0	0	1
Hematological system disease (coagulation disorders)	0	0	1	0	0
Others (stomatitis, polyserositis, ascites, cytomegalovirus infection)	0	3	0	0	1

This study analyzed the proportion of the number of hospitalizations for the corresponding disease in the total number of hospitalizations between 2016 and 2021. The hospitalization ratio of EBV-LPD was 20.06/1 000 000 hospitalized children (153/7 626 411), of which EBV^+^ T-LPD cases accounted for the largest proportion with 13.11/1 000 000 hospitalized children (100/7 626 411) (Supplementary Fig. 1). This study further analyzed the evolving trends in hospitalizations, expenditures, and LOS for the five EBV-LPDs from 2016 to 2021, as shown in Supplementary Fig. 2. It is evident that hospitalizations for EBV^+^ T-LPD has increased since 2020. Moreover, begining in 2020, the overall economic burden of these five diseases has increased in comparison to 2019. The specific characteristics of each disease are described below.

### T- and NK-cell associated EBV-LPD

#### EBV^+^ T-LPD

**Fig. 1:**
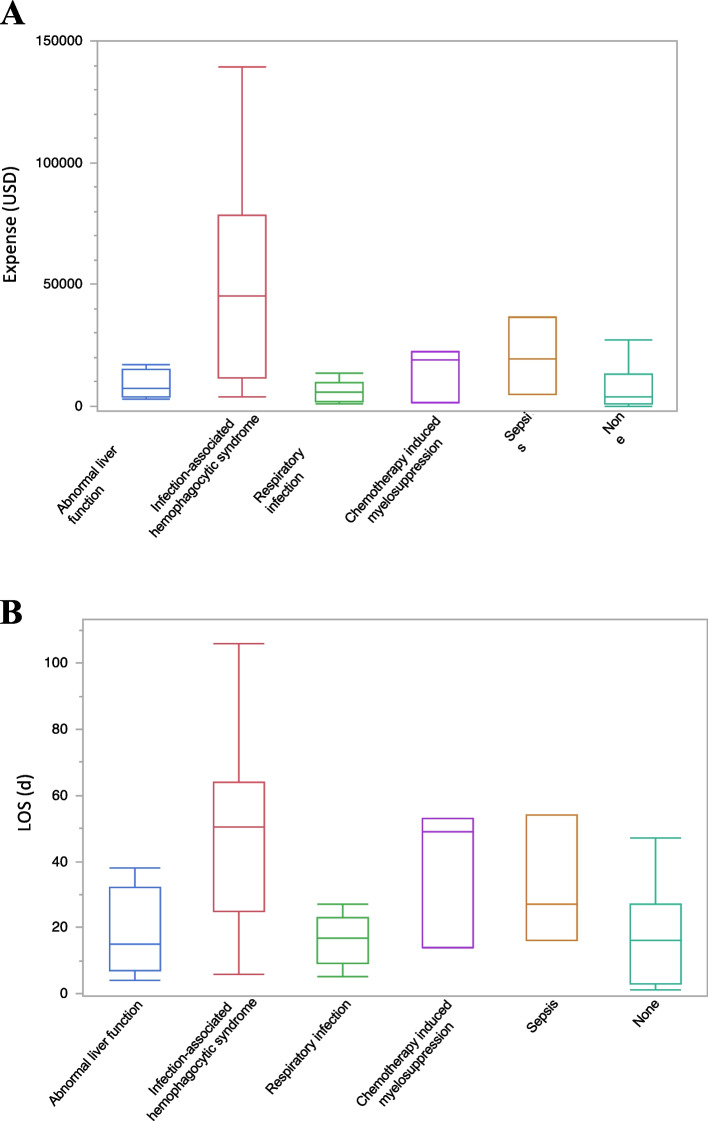
LOS and expenses of different complications in children hospitalized for EBV^+^ T-LPD. **A** The expenses of different complications. **B** The LOS of different complications

By analyzing the FSMRs of children hospitalized with EBV^+^ T-LPD, we found that a total of 100 patients were diagnosed with EBV^+^ T-LPD, accounting for 0.013‰ (100/7 626 411) and 65.36% of the total and EBV-LPDs hospitalizations of patients, respectively. As shown in Table 2, the male-to-female ratio of EBV^+^ T-LPD children is 1.04:1, school-age children (6–12 y, 57.00%) accounted for greater than 50%. There was no obvious seasonality in hospitalization rates for EBV^+^ T-LPD (Supplementary Fig. 3). We analyzed the demographic characteristics of children with EBV^+^ T-LPD in different places of residence (urban/rural) (Table 4), the results show that 64.00% (64/100) of children with EBV^+^ T-LPD lived in rural areas, while no statistical differences were noted among the subgroups of gender, age, ethnicity, years of admission, LOS, etc. Complications occurred in 49% (49/100) of EBV^+^ T-LPD cases, mainly including hemophagocytic lymphohistiocytosis (HLH) (22.00%), abnormal liver function (12.00%), respiratory infection (9.00%), chemotherapy induced myelosuppression (3.00%) and sepsis (3.00%). Among them, children with EBV^+^ T-LPD complicated by HLH have the longest LOS and the highest expenses (Fig. 1). Moreover, the average LOS for children with HLH was 50.6 d and an average hospitalization cost of 323 383.51 USD, both significantly surpassing those of children with other complications (Supplementary Fig. 4). One death due to EBV^+^ T-LPD was reported in FSMRs. This patient is a 12-year-old female living in a rural area who developed HLH and disseminated intravascular coagulation during hospitalization.

**Table 4:** The general socio-demographic characteristics and disease burden of EBV^+^ TLPD categorized by residence during pediatric inpatient hospitalizations from January 2016 to December 2021

Categories	Residence		χ^2^ value	*p* value
	**Urban**	**Rural**		
**Overall (n, %)**	36 (36.00)	64 (64.00)	-	-
** Gender (n, %)**				
Male	19 (38.78)	30 (61.22)	0.321	0.5709
Female	17 (33.33)	34 (66.67)		
**Age (n, %)**				
29 d– < 1 y	3 (60.00)	2 (40.00)	-	0.5928
1–3 y	1 (14.29)	6 (85.71)		
4–6 y	5 (31.25)	11 (68.75)		
6–12 y	22 (38.60)	35 (61.40)		
13–18 y	5 (33.33)	10 (66.67)		
**Ethnicity (n, %)**				
Han	33 (34.74)	62 (65.26)	1.316	0.2513
Non-Han	3 (60.00)	2 (40.00)		
**Region (n, %)**				
North China	18 (27.69)	47 (72.31)	5.813	0.0547
East China	6 (46.15)	7 (53.85)		
Central China	12 (54.55)	10 (45.45)		
**Year of admission (n, %)**				
2016	2 (28.57)	5 (71.43)	-	0.6047
2017	2 (28.57)	5 (71.43)		
2018	1 (16.67)	5 (83.33)		
2019	1 (14.29)	6 (85.71)		
2020	12 (36.36)	21 (63.64)		
2021	18 (45.00)	22 (55.00)		
**Complication (n, %)**				
Abnormal liver function	4 (33.33)	8 (66.67)	-	0.3212
Infection-associated hemophagocytic syndrome	9 (40.91)	13 (59.09)		
Respiratory infection	3 (33.33)	6 (66.67)		
Chemotherapy induced myelosuppression	1 (33.33)	2 (66.67)		
Sepsis	3 (100.00)	0 (00.00)		
None	16 (31.37)	35 (68.63)		
** LOS [d, median (IQR)]**	18 (10.25–34.5)	21 (9.25–38)	0.0579	0.8098
** Expense [USD, median (IQR)]**	7739.72 (3012.23–33,745.54)	6451.57 (2282.75–21,894.35)	0.2315	0.6304

*EBV*^+^*TLPD* EBV positive T-cell lymphoproliferative disease, *LOS* Length of stay, *IQR* Inter quartile range, *USD* USA dollar

#### ENKTL-NT

The 27 ENKTL-NT cases were reported in FSMRs, accounting for 0.004‰ (27/7 626 411) and 17.65% (27/153) of the total and EBV-LPDs hospitalizations, respectively. As Table 2 shows, the school-age children group (6–12 y; 51.85%) accounted for the highest proportion. The proportion of children living in rural areas (77.78%, 21/27) was higher than the proportion of children living in urban areas (22.22%, 6/27). Among 27 children with ENKTL-NT, 21 had complications (77.78%, 21/27). The main complications were HLH (22.22%), respiratory diseases (18.52%), and chemotherapy induced myelosuppression (11.11%). Table S1 shows the general socio-demographic characteristics and disease burden of 27 children with ENKTL-NT.

#### CSEBV^+^ T-LPD

We found a total of 11 children diagnosed with CSEBV^+^ T-LPD (Table S2), accounting for 0.001‰ (11/7 626 411) and 7.19% (11/153) of the total and EBV-LPDs hospitalizations, respectively. The male-to-female ratio of CSEBV^+^ T-LPD children was 1.75:1. School-age group (6–12 y) have the largest number of cases, accounting for 36.36%. The median LOS was 14 days (IQR 8–33), and the median expense was 7 400.09 USD (IQR 2 806.81–33 536.52). Complications occurred in 9 of the 11 children, and 6 of them were complicated by HLH (54.55%).

#### NKTL

We counted a total of 6 NKTL cases, accounting for 0.0008‰ (6/7 626 411) and 3.92% (6/153) of the total and EBV-LPD hospitalizations, respectively (Table S3). The median age of onset of NKTL was 12.5 years (IQR 9–13.75). One-third of cases were complicated by HLH. The median LOS of the children was 26.5 days (IQR 3–42), and the median expense was 8 833.31 USD (IQR 1 104.24–26 001.48).

### Immunodeficiency related EBV-LPD

#### PTLD

Finally, we analyzed 9 PTLD cases reported in FSMRs (Table S4). The primary diseases of these children include leukemia (33.33%, 3/9), thalassemia (22.22%, 2/9), aplastic anemia (22.22%, 2/9), and Hodgkin disease (11.11%, 1/9), etc. (Supplementary Fig. 5). Among the 9 patients, 8 patients received hematopoietic stem cell transplantation and only 1 patient received liver transplantation. The results showed that PTLD accounted for 0.001‰ (9/7 626 411) of the total number of hospitalized children and 5.88% (9/153) of the hospitalizations for EBV-LPD, among which the 6–12 y group accounted for the highest proportion (55.56%, 5/9), followed by the 13–18 y group (22.22%, 2/9) (Table 2). Subsequently, we analyzed the complications of PTLD and found that the most common complications were respiratory infection (44.44%, 4/9) (Table S4).

## Discussion

EBV-LPDs include non-neoplastic and neoplastic lymphoproliferative diseases, most cases have an acute onset and severe symptoms, which can easily cause different complications [14]. This study summarized and analyzed the FSMRs of hospitalized children diagnosed with five types of EBV-LPDs in China from 2016 to 2021.

EBV^+^ T-LPD and CSEBV^+^ T-LPD can occur after primary EBV infection. These diseases are common in children and adolescents aged 15 to 20 y [15]. The results of our study show that EBV^+^ T-LPD mostly occurs in school-age children, and the proportion of boys and girls is almost equal. It has been reported that CSEBV^+^ T-LPD has an extremely high mortality rate due to its explosive and extremely short course [16]. However, in this study, there were no deaths among children with CSEBV^+^ T-LPD, while there was only one death case among children with EBV^+^ T-LPD. The possible reasons are that firstly, the total number of disease cases is small, and secondly, due to limited FSMRs information, we were unable to track the outcomes of children. A single-center retrospective study on CSEBV^+^ T/NK-LPDs conducted in southwest China showed that 44% of CSEBV^+^ T/NK-LPD cases were complicated with HLH [17]. In our study, CSEBV^+^ T-LPD cases with HLH accounted for 54.55%, which is almost consistent with the finding of the aforementioned single-center study.

NKTL and ENKTL-NT are subtypes of EBV-related non-Hodgkin lymphoma. Our study suggests that ENKTL-NT are more common in boys, which is consistent with previous studies [8]. Both diseases mostly occur in rural areas, which is consistent with previous studies reporting that rural populations are susceptible to such diseases [18]. This may be related to environmental and lifestyle factors in rural areas, including infectious microorganisms, solvents, drugs and other chemicals [19]. We found that, both NKTL and ENKTL-NT are more common in children over school age, less common in infants and younger children. In a retrospective study report on ENKTL-NT, it was found that 11.3% (23/203) of ENKTL-NT children were complicated by HLH [20], while in our study, 22.22% of the children were complicated by HLH. This variance could potentialy be attributed to following reasons. Firstly, the subjects in the aforementioned study comprised adults and children, whereas our study focus on children. Secondly, it is plausible that the incidence of HLH in ENKTL-NT differ between adults and children, leading to variations in complication rates observed between the two studies.

Previous studies have reported that among tumors, lymphoma is the most common triggering factor for HLH [21], with T cell lymphoma and NK cell lymphoma being the most common [22]. Our study also found that a certain proportion of cases among the T- and NK-cell associated EBV-LPDs are complicated by HLH, and this complication is directly related to the higher economic burden of disease and increased LOS in children with EBV-LPDs. Furthermore, by comparing the LOS and hospitalization costs of patients with and without HLH, we found that the increase in disease burden was mainly caused by prolonged hospitalization.This suggests that clinicians should pay great attention to the possibility of children's condition developing into HLH, closely monitor the children's condition, and actively prevent and treat it.

PTLD is one of the most fatal complications after solid stem cell transplantation and allogeneic hematopoietic stem cell transplantation, which will seriously affect the survival rate of children. Studies have reported that EBV infection is the main cause of PTLD, and about 50–70% of PTLD biopsies are EBV positive [23]. In our study, most PTLD cases were positive for EBV, which to a certain extent suggests the epidemiological characteristics of hospitalized children with EBV-PTLD. A single-center study reported that the primary cause of death among PTLD patients was infection. Additionally, the study found that the one-year mortality rate for PTLD patients was 25%, with 48% of these deaths attributed to infection [24]. Consistent with these finding, respiratory infection emerged as the most common complication among children with PTLD in our study. This highlights critical importance of ongoing surveillance for pathogenic infections and early detection to improve patient outcomes. While various complications occurred in these cases, it's important to note that no deaths were recorded in the database because we were unable to track the outcome of the children.

In addition, we compiled and analyzed data on the changing trends in the number of hospitalizations, expenses, and LOS for EBV-LPDs from 2016 to 2021. We found that hospitalizations for EBV^+^ T-LPD increased during the years. Moreover, since the outbreak of COVID-19, the overall economic burden of these diseases has increased compared with 2019. Nevertheless, we cannot yet definitively determine the correlation between COVID-19 and the disease burden of patients hospitalized for EBV-LPDs.

There are some limitations to this study. 1) Although these 5 diseases have been confirmed to be highly related to EBV, the exact relationship between some cases and EBV has not been described in FSMRs. Nonetheless, our data can still provide some insights into the epidemiological characteristics of hospitalized children with EBV-LPD. 2) Since FSMRs only contain basic medical information about children, we are unable to obtain comprehensive laboratory results, clinical data and follow-up data. 3) Due to differences in the classification and naming of EBV-LPD between the ICD-10 code and WHO, based on the existing database, we can only retrieve 5 types of EBV-LPDs based on the ICD-10 code, which means some EBV-LPDs have not been documented. For example, chronic active Epstein-Barr virus infection (CAEBV) is defined by WHO as an EBV-LPD, but there is no diagnostic name for CAEBV in the ICD-10 code [25], so it was not included in this study, but it is speculated that most of the non-neoplastic EBV^+^ T-LPD cases in children are CAEBV, which can reflect the epidemiological characteristics of CAEBV to some extent.

## Conclusions

Compared to the total number of children hospitalized in China within the same age group and time period, the ratio of EBV-LPD is found to be relatively low. However, EBV-LPD can affect individuals of all age groups, with a higher prevalence among school-age children. Additionally, the ratio of occurrence between boys and girls is almost equal. Among the 5 types of EBV-LPDs studied, EBV^+^ T-LPD has the highest proportion. Moreover, apart from PTLD, the remaining 4 types of EBV-LPDs tend to occur more frequently in rural areas than in urban areas. Despite the low incidence rate, the overall disease economic burden of EBV-LPD is relatively high. HLH is a common complication, and its presence can directly affect the disease burden experienced by patients because of prolonged hospitalization. This study analyzed the FSMRs of hospitalized children with EBV-LPD collected by 27 hospitals in different regions of China from 2016 to 2021, and summarized their epidemiological characteristics, disease expense, complications and other aspects, which enriched the existing epidemiological and disease burden content of EBV-LPDs. It also contributed to the rational allocation of healthcare resources, and provide a foundation for the development of relevant treatment strategies.

### Supplementary Information

Supplementary Material 1. The proportion of patients hospitalized for EBV-LPDs from January 2016 to December 2021

Supplementary Material 2. The changing trends in the number of hospitalizations, expenses, and LOS for EBV-LPDs from 2016 to 2021

Supplementary Material 3. Monthly cases and proportions of children hospitalized for EBV-LPD from January 2016 to December 2021. M(n): number of monthly hospitalizations, Y(n): number of yearly hospitalizations. The blue bars represent the ratio of M(n)/Y(n), and the orange line represents M(n). The color version of this figure is available in the online edition

Supplementary Material 4. Comparison of average expenses and LOS between EBV^+^ T-LPD hospitalized children with and without HLH

Supplementary Material 5. The distribution of primary disorders in children hospitalized with PTLD

Supplementary Material 6.

## Data Availability

The data generated during the current study are available from the corresponding author on a reasonable request.

## Abbreviations

LPDs: Lymphoproliferative disorders
EBV: Epstein-Barr virus
NK: Natural killer
EBV-LPDs: EBV-associated lymphoproliferative disorders
EBV^+^ T-LPD: EBV-positive T-cell lymphoproliferative disease
NKTL: NK/T cell lymphoma
ENKTL-NT: Extranodal NK/T-cell lymphoma (nasal type)
CSEBV^+^ T-LPD: Systemic EBV-positive T-cell lymphoproliferative disease of childhood
PTLD: Posttransplant lymphoproliferative disorders
alloHSCT: Allogeneic hematopoietic stem cell transplantation
FRCPD: The Futang Research Center of Pediatric Development
FSMRs: The face sheet of discharge medical records
FUTURE: The FUTang Update medical REcords
LOS: Length of stay
IQR: Interquartile ranges
USD: USA dollar
HLH: Hemophagocytic lymphohistiocytosis
CAEBV: Chronic active Epstein-Barr virus infection
