# Supplementary figures and images for: Clinical epidemiology of Epstein-Barr virus-associated Lymphoproliferative Disorders (EBV-LPDs) in hospitalized children: A six-year multi-institutional study in China
Source: Ital J Pediatr. 2024 Jul 2;50:125. doi: 10.1186/s13052-024-01685-y (PMC11218373; doi:10.1186/s13052-024-01685-y)

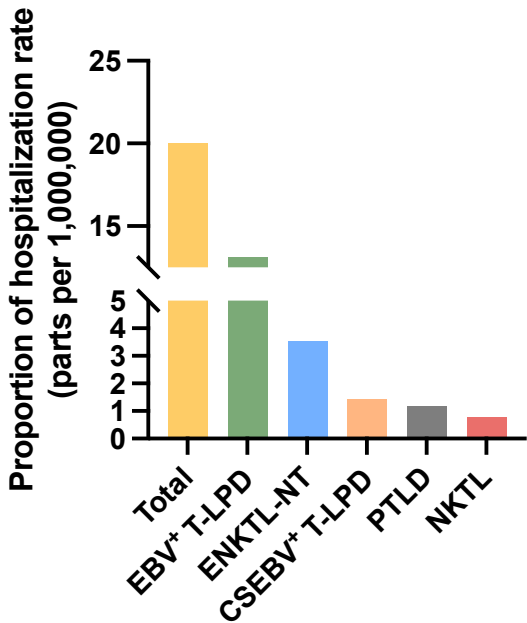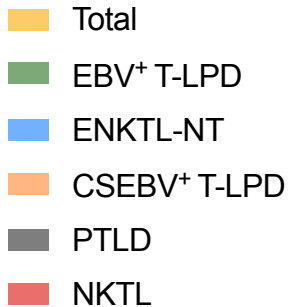

Supplement: Supplementary file 1 — Supplementary Material 1. The proportion of patients hospitalized for EBV-LPDs from January 2016 to December 2021 [file 13052_2024_1685_MOESM1_ESM.pdf]

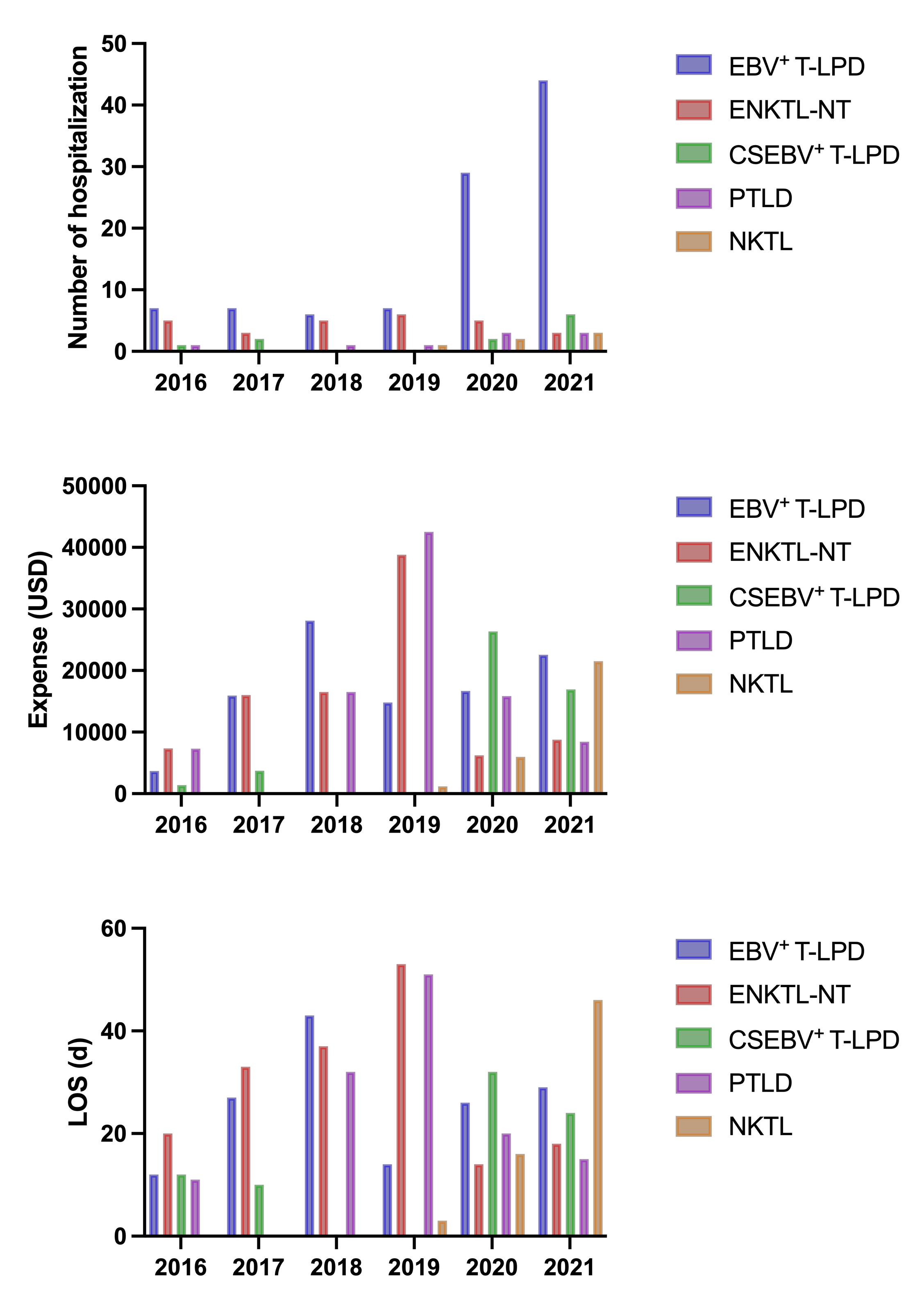

Supplement: Supplementary file 2 — Supplementary Material 2. The changing trends in the number of hospitalizations, expenses, and LOS for EBV-LPDs from 2016 to 2021 [file 13052_2024_1685_MOESM2_ESM.tiff]

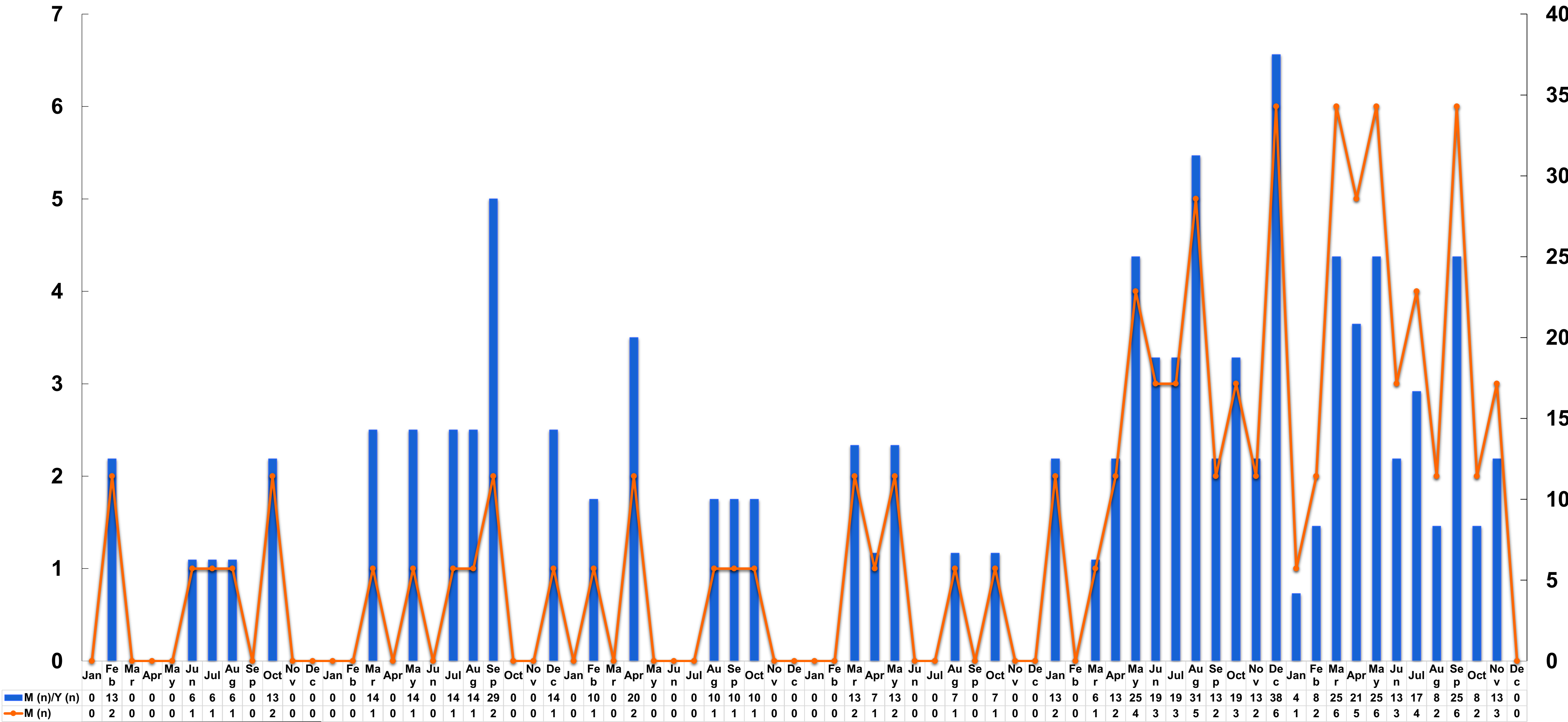

Supplement: Supplementary file 3 — Supplementary Material 3. Monthly cases and proportions of children hospitalized for EBV-LPD from January 2016 to December 2021. M(n): number of monthly hospitalizations, Y(n): number of yearly hospitalizations. The blue bars represent the ratio of M(n)/Y(n), and the orange line represents M(n). The color version of this figure is available in the online edition [file 13052_2024_1685_MOESM3_ESM.pdf]

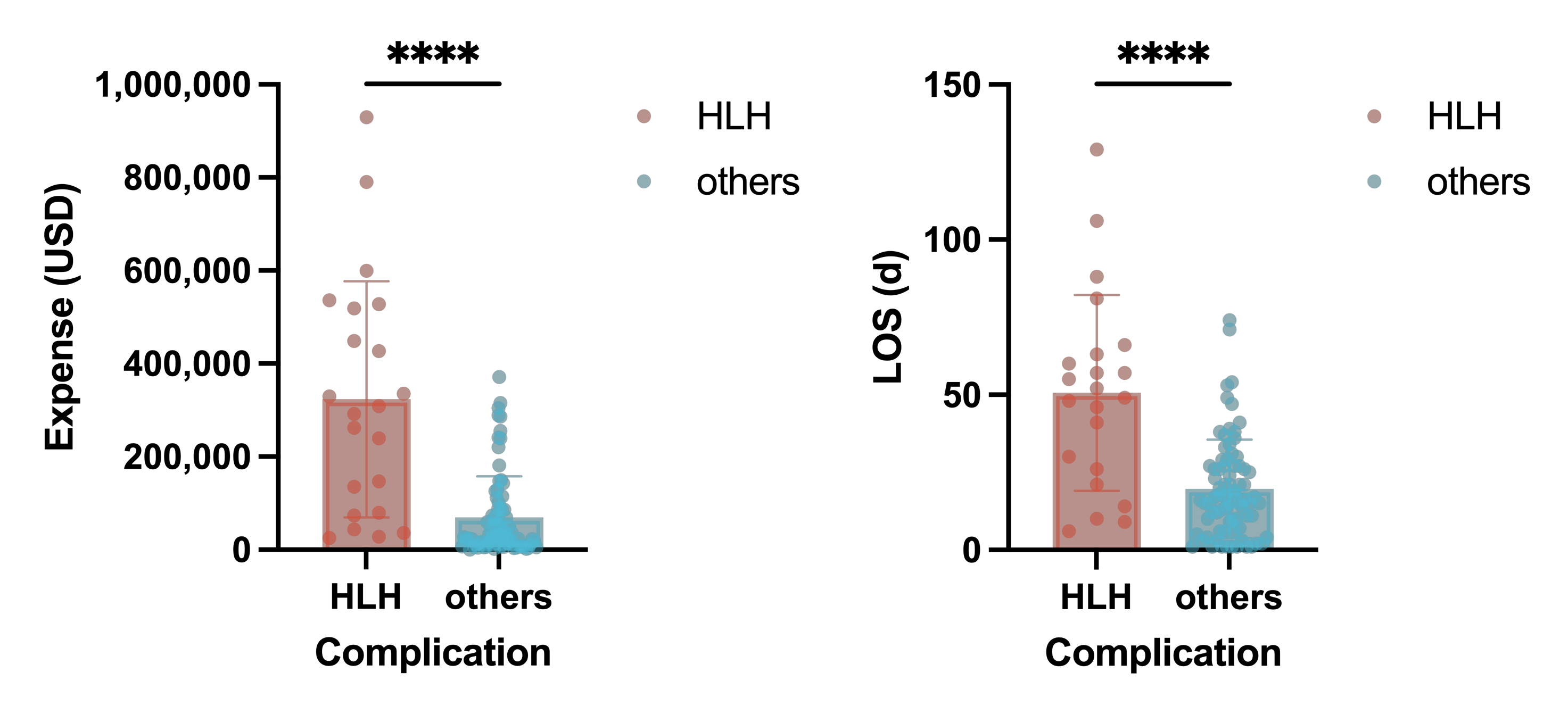

Supplement: Supplementary file 4 — Supplementary Material 4. Comparison of average expenses and LOS between EBV+ T-LPD hospitalized children with and without HLH [file 13052_2024_1685_MOESM4_ESM.tiff]

# CASE NUMBER

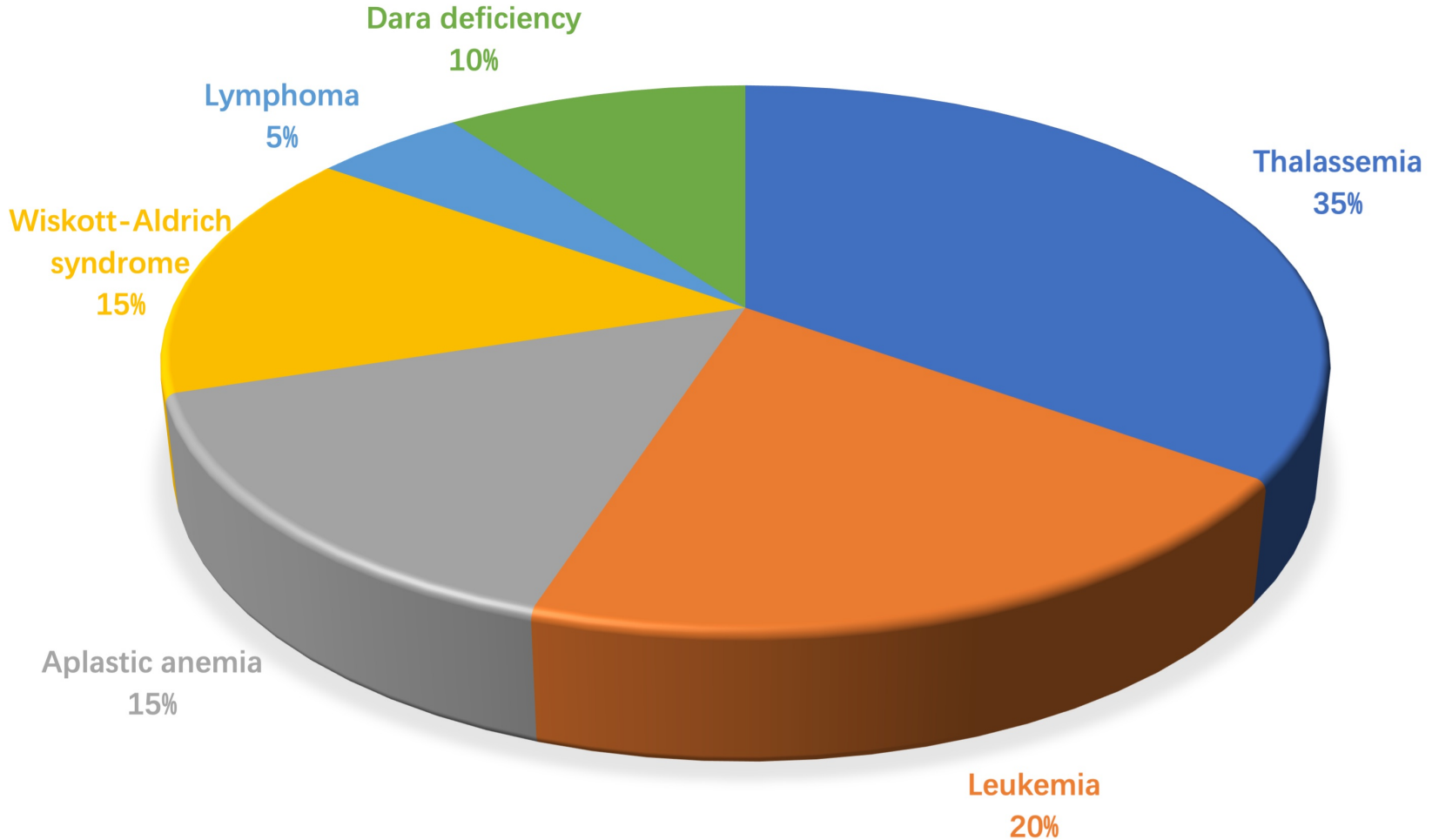

Supplement: Supplementary file 5 — Supplementary Material 5. The distribution of primary disorders in children hospitalized with PTLD [file 13052_2024_1685_MOESM5_ESM.pdf]
