# Supplementary material for: Clinical epidemiology of Epstein-Barr virus-associated Lymphoproliferative Disorders (EBV-LPDs) in hospitalized children: A six-year multi-institutional study in China
Source: Ital J Pediatr. 2024 Jul 2;50:125. doi: 10.1186/s13052-024-01685-y (PMC11218373; doi:10.1186/s13052-024-01685-y)
Supplement: Supplementary file 6 — Supplementary Material 6. [file 13052_2024_1685_MOESM6_ESM.docx]

**TABLE S1 The general socio-demographic characteristics and disease burden of ENKTL-NT during impatient hospitalizations form January 2016 to December 2021**

| **No.** | **Gender** | **Age (years)** | **Province (Region of China)** | **Ethnicity** | **Residence** | **Complications** | **LOS (d)** | **Expense (USD)** |
| --- | --- | --- | --- | --- | --- | --- | --- | --- |
| **1** | Female | 12 | Henan (Central) | Han | Rural | Hemophagocytic syndrome | 34 | 32 706.75 |
| **2** | Female | 14 | Liaoning (Northeast) | Han | Rural | Hemophagocytic syndrome | 32 | 15 855.41 |
| **3** | Female | 6 | Jiangsu (East) | Han | Urban | Hemophagocytic syndrome | 7 | 3 873.62 |
| **4** | Female | 6 | Shandong (East) | Han | Urban | Chemotherapy induced myelosuppression | 34 | 30 256.56 |
| **5** | Male | 4 | Shandong (East) | Han | Rural | Acute bronchitis | 38 | 17 802.07 |
| **6** | Male | 13 | Anhui (East) | Han | Urban | Sepsis | 4 | 2 305.46 |
| **7** | Female | 13 | Jiangsu (East) | Han | Urban | Liver damage | 24 | 8 877.75 |
| **8** | Female | 8 | Guangdong (South) | Han | Urban | Thrombotic microangiopathy | 177 | 117 402.2 |
| **9** | Male | 10 | Inner Mongolia (North) | Han | Urban | Stomatitis | 27 | 7 811.41 |
| **10** | Male | 7 | Inner Mongolia (North) | Han | Rural | -^1^ | 7 | 2 546.32 |
| **11** | Female | 3 | Hubei (Central) | Han | Rural | Hemophagocytic syndrome | 53 | 42 008.55 |
| **12** | Male | 8 | Hebei (North) | Han | Rural | Chemotherapy induced myelosuppression | 15 | 9 912.07 |
| **13** | Female | 12 | Inner Mongolia (North) | Han | Rural | Chemotherapy induced myelosuppression | 30 | 8 991.76 |
| **14** | Female | 1 | Shanxi (North) | Han | Rural | Bronchopneumonia | 51 | 18 236.04 |
| **15** | Male | 4 | Liaoning (North) | Han | Rural | Bronchopneumonia | 19 | 11 952.26 |
| **16** | Male | 12 | Henan (Central) | Han | Rural | Respiratory disease | 12 | 862.53 |
| **17** | Male | 1 | Anhui (East) | Han | Rural | Severe pneumonia | 10 | 4 839.94 |
| **18** | Female | 12 | Anhui (East) | Han | Rural | Sepsis | 55 | 45 303.35 |
| **19** | Male | 12 | Shandong (East) | Han | Rural | Myocardial strain | 11 | 4 555.30 |
| **20** | Male | 11 | Hebei (North) | Non-Han | Rural | Hemophagocytic syndrome | 30 | 21 239.79 |
| **21** | Male | 7 | Anhui (East) | Han | Rural | Polyserositis | 14 | 8 049.77 |
| **22** | Male | 16 | Qinghai (Northwest) | Non-Han | Rural | Ascites | 28 | 10 517.8 |
| **23** | Female | 15 | Hebei (North) | Han | Rural | Hemophagocytic syndrome | 101 | 25 513.35 |
| **24** | Male | 14 | Shandong (East) | Han | Rural | -^1^ | 9 | 2 488.36 |
| **25** | Male | 10 | Jiangsu (East) | Han | Rural | -^1^ | 1 | 2 175.19 |
| **26** | Male | 12 | Inner Mongolia (North) | Han | Rural | -^1^ | 3 | 1 064.06 |
| **27** | Male | 12 | Henan (Central) | Han | Rural | -^1^ | 3 | 708.056 |
| **Median (IQR)** | - | 11 (6.5–12) | - | - | - | - | 24 (9–34) | 8 991.76 (2 546.32–21 239.79) |

ENKTL: extranodal NK/T cell lymphoma, nasal type

LOS: length of stay

IQR: inter quartile range

USD: USA dollar

^1^data deficient

**TABLE S2 The general socio-demographic characteristics and disease burden of CSEBV^+^ T-LPD during impatient hospitalizations form January 2016 to December 2021**

| **No.** | **Gender** | **Age (years)** | **Province (Region of China)** | **Ethnicity** | **Residence** | **Complication** | **History of blood transfusion** | **LOS (d)** | **Expense (USD)** |
| --- | --- | --- | --- | --- | --- | --- | --- | --- | --- |
| **1** | Male | 7 | Anhui  (East) | Han | Urban | Hemophagocytic syndrome | Yes | 13 | 9 794.24 |
| **2** | Male | 5 | Shandong  (East) | Han | Urban | Hemophagocytic syndrome | Yes | 16 | 7 152.59 |
| **3** | Female | 10 | Zhejiang  (East) | Han | Urban | Respiratory failure | Yes | 30 | 38 689.13 |
| **4** | Female | 7 | Jiangxi  (East) | Han | Urban | Acute pharyngitis | No | 3 | 306.42 |
| **5** | Male | 14 | Anhui  (East) | Han | Rural | Hemophagocytic syndrome | No | 8 | 7 400.09 |
| **6** | Female | 1 | Liaoning  (Northeast) | Han | Rural | Hemophagocytic syndrome | Yes | 14 | 7 005.18 |
| **7** | Male | 13 | Hebei  (North) | Han | Rural | Hemophagocytic syndrome | No | 5 | 2 806.81 |
| **8** | Female | 13 | Yunnan  (Southwest) | Han | Rural | Hemophagocytic syndrome | Yes | 57 | 33 536.52 |
| **9** | Male | 4 | Liaoning  (Northeast) | Han | Rural | Coagulation disorders | Yes | 44 | 41 130.01 |
| **10** | Male | 2 | Jiangxi  (East) | Han | Rural | Pulmonary infection | No | 33 | 14 053.24 |
| **11** | Male | 9 | Shaanxi  (Northwest) | Han | Rural | -^1^ | No | 12 | 1 383.54 |
| **Median (IQR)** | - | 7 (4.5–11.5) | - | - | - |  | - | 14 (8–33) | 7 400.09 (2 806.81–33 536.52) |

CSEBV^+^ T-LPD: systemic EBV-positive T-cell lymphoproliferative disease of childhood

LOS: length of stay

IQR: inter quartile range

USD: USA dollar

^1^data deficient

**TABLE S3 The general socio-demographic characteristics and disease burden of NKTL during impatient hospitalizations form January 2016 to December 2021**

| **No.** | **Gender** | **Age (years)** | **Province (Region of China)** | **Ethnicity** | **Residence** | **Complication** | **History of blood transfusion** | **LOS (d)** | **Expense (USD)** |
| --- | --- | --- | --- | --- | --- | --- | --- | --- | --- |
| **1** | Female | 14 | Shanxi  (North) | Han | Urban | Hemophagocytic syndrome, respiratory disease | Yes | 25 | 6 556.86 |
| **2** | Female | 7 | Heilongjiang  (Northeast) | Han | Urban | Gastrointestinal bleeding | Yes | 3 | 841.95 |
| **3** | Male | 13 | Shanxi  (North) | Han | Rural | Hemophagocytic syndrome, pneumonia | Yes | 28 | 11 109.76 |
| **4** | Male | 16 | Qinghai  (Northwest) | Non-Han | Rural | Chemotherapy induced myelosuppression, severe pneumonia | Yes | 28 | 23 028.83 |
| **5** | Male | 8 | Yunnan  (Southwest) | Han | Rural | Sepsis, severe pneumonia | Yes | 84 | 34 919.44 |
| **6** | Female | 12 | Henan  (Central) | Han | Rural | -^1^ | No | 3 | 1 191.68 |
| **Median (IQR)** | - | 12.5 (9–13.75) | - | - | - |  | - | 26.5 (3–42) | 8 833.31 (1 104.24–26 001.48) |

NK/TCL: NK/T cell lymphoma

LOS: length of stay

IQR: inter quartile range

USD: USA dollar

^1^data deficient

**TABLE S4 The general socio-demographic characteristics and disease burden of PTLD during impatient hospitalizations form January 2016 to December 2021**

| **No.** | **Gender** | **Age (years)** | **Province (Region of China)** | **Ethnicity** | **Residence** | **Complication** | **History of blood transfusion** | **LOS (d)** | **Expense (USD)** |
| --- | --- | --- | --- | --- | --- | --- | --- | --- | --- |
| **1** | Female | 12 | Shanxi (North) | Han | Urban | Thrombotic microangiopathy | Yes | 51 | 42 530.42 |
| **2** | Male | 13 | Beijing (North) | Han | Urban | Severe pneumonia | Yes | 33 | 33 549.69 |
| **3** | Male | 10 | Shandong (East) | Han | Urban | Cytomegalovirus infection | No | 21 | 10 785.74 |
| **4** | Female | 8 | Guangdong (South) | Han | Urban | Sepsis | No | 19 | 8 717.72 |
| **5** | Male | 7 | Guangdong (South) | Han | Urban | Intestinal infection | No | 11 | 5 073.37 |
| **6** | Male | 13 | Inner Mongolia (North) | Han | Rural | Trachitis | No | 16 | 11 520.73 |
| **7** | Male | 1 | Guangdong (South) | Han | Rural | Pneumonia | No | 7 | 3 221.19 |
| **8** | Female | 2 | Hubei (Central) | Han | Rural | Pulmonary fungal infection | No | 32 | 16 531.18 |
| **9** | Female | 8 | Shanxi (North) | Han | Rural | None | Yes | 11 | 7 329.38 |
| **Median (IQR)** | - | 8 (7–12) | - | - | - |  | - | 19 (11–32) | 10 785.74  (7 329.38–16 531.18) |

PTLD: posttransplant lymphoproliferative disorders

LOS: length of stay

IQR: inter quartile range

USD: USA dollar
